# Nurses’ Experiences Concerning Older Adults with Polypharmacy: A Meta-Synthesis of Qualitative Findings

**DOI:** 10.3390/healthcare11030334

**Published:** 2023-01-23

**Authors:** Cheng Cheng, Huan Yu, Qingling Wang

**Affiliations:** 1 School of Nursing, Fudan University, Shanghai 200032, China; 2 School of Nursing, Anhui Medical University, Hefei 230032, China; 3 School of Nursing and Health Management, Shanghai University of Medicine and Health Sciences, Shanghai 201318, China

**Keywords:** nurse, older adults, polypharmacy, thematic synthesis

## Abstract

Polypharmacy is an increasing health concern among older adults and results in many health risks. Nurses have an important role to play in supporting medication management and promoting medication safety across different settings. This study aims to provide a meta-synthesis of qualitative studies investigating the perceptions and experiences of nurses in caring for older adults with polypharmacy. Electronic databases including PsycArticles, CINAHL Complete, MEDLINE, and ERIC were searched between September 2001 and July 2022. Potential studies were checked against inclusion and exclusion criteria. We included peer-reviewed studies reporting data on the experiences of nursing staff across different settings. Studies unitizing any qualitative approach were included, and the included studies were reviewed and analyzed using a thematic synthesis approach. Study quality was examined using the Critical Appraisal Skills Programme checklist for qualitative research. A total of nine studies with 91 nurses were included. Four major themes emerged: older adults suffering from polypharmacy, the importance of multidisciplinary teams, nursing roles in caring for older adults, and the complexity and barriers of implementing polypharmacy management. Healthcare professionals should pay attention to the impacts of polypharmacy in older adults’ lives and should acknowledge the importance of team-based polypharmacy care in supporting older adults. Nurses play a key role in caring for older adults with polypharmacy, therefore, they should be empowered and be involved in medication management.

## 1. Introduction

Due to rising health issues in older adults, polypharmacy has become more common. The term polypharmacy can be defined either quantitatively (multiple medications) or qualitatively (unnecessary prescriptions) [1]. The phenomenon of polypharmacy (simultaneous prescription of ≥5 medicines) is common both in clinical and community settings, with a reported prevalence among older adults of approximately 50.1% in China [2], 65.1% in the United States [3], and ranging from 26.3% to 39.9% in European countries [4].

Although using multiple medicines or polypharmacy may be clinically appropriate in many cases, the negative clinical consequences of polypharmacy have been reported. Older people with polypharmacy may have a higher likelihood of drug–drug interactions [5] and adverse drug-related events [6]. Research has also linked polypharmacy with frailty, repeated hospitalizations, cognitive impairment, reduced physical function, and mortality [7]. Qualitative evidence showed that polypharmacy might pose a high burden on older people’s lives and result in negative feelings. For example, Eriksen, et al. [8] found that older adults might experience challenges and barriers to managing polypharmacy, and that having polypharmacy adversely affects their quality of life and adherence.

Nurses play an important role in helping to identify people with polypharmacy and facilitating risk reduction [9]. An integrative review identified three features regarding the role of nurses during medication management of transitional care: (1) implementation of medication reconciliation, (2) collaborating with other health care professionals, and (3) supporting health care recipients [10]. Recent evidence has shown that nurse-led programs might contribute to successful medicine management and a reduction in medication errors. For example, Yang et al. [11] conducted a randomized controlled trial involving 136 older adults with multimorbidity and found that the intervention group reported having more adherence to medication, self-efficacy, knowledge about the management and harm of medication, and greater satisfaction with medication use.

Qualitative research is suggested as an appropriate research design to provide insights into particular phenomena. The output of qualitative research may help inform the effectiveness, feasibility, and acceptability of nursing practice [12]. Given that health care professionals’ experiences, attitudes, and perceptions of polypharmacy may impact patient management [13], an increasing number of studies have been conducted within the area of polypharmacy from the perspectives of general practitioners [14], pharmacists [15] and mixed health care professionals [16,17]. However, evidence documenting nurses’ experiences with polypharmacy is scarce. Thus, the purpose of this study is to collect, critically appraise, synthesize, and present existing studies on the polypharmacy-related experiences of nurses.

## 2. Materials and Methods

The report of this synthesis adhered to the enhancing transparency in reporting the synthesis of qualitative research (ENTREQ) statement [18].

### 2.1. Search Strategy

An electronic search of APA PsycArticles, CINAHL Complete, MEDLINE, and ERIC was conducted between September 2001 and July 2022 with keywords and index terms used to describe the studies. The search strategy was adapted for each data source, and an additional search was conducted before the submission of this review. The electronic search was complemented by tracking reference lists of the most relevant reviews and expert counselling in the region. We adapted the search strategy to meet the requirements of different databases. To ensure a comprehensive search, no search filters were used.

A full search strategy was presented below as an example:

DatabaseCINAHL Complete via EBSCO PlatformInitial date of search1 October 2021Additional date of search20 July 2022

Search strategy: (polypharmacy or multiple drug* (* is for search convenience as so as to include all variations of a word in the search.) or multiple medication* or many drug* or many medication*) AND (nurse or nurses or nursing or nursing staff or registered nurse) AND (qualitative research or qualitative study or qualitative methods or interview or ethnographic or phenomenological or case study) AND (older adults or elderly or geriatric or geriatrics or aging or senior or seniors or older people or aged 65 or 65+).

### 2.2. Inclusion and Exclusion Criterion

This review considered primary studies that (1) involved nursing staff across health care settings, (2) drew on experiences, perceptions, and attitudes regarding polypharmacy among older adults (people at least 65 years of age), (3) adopted qualitative designs including, but not limited to, grounded theory, phenomenology, and action research, (4) were reported in English or Chinese, and (5) were published in peer-reviewed journals.

### 2.3. Study Selection

All the citations from the search were transferred into Endnote X9 (Clarivate Analytics, Philadelphia, PA, USA) and duplicates were removed. Two reviewers screened the titles and abstracts of those citations based on the inclusion criteria and retrieved the full text of potential studies. The reviewers then checked the full-text studies against the inclusion and exclusion criteria.

### 2.4. Quality Assessment

Two reviewers critically appraised the data quality of the included studies using the Critical Appraisals Skills Programme (CASP) for qualitative research [19]. Any disagreement regarding the quality assessment was discussed until a consensus was reached.

### 2.5. Data Extraction and Synthesis

The data extracted included the phenomena of interest, research settings, participants’ characteristics, study methods, key findings, and related illustrations.

The extracted qualitative findings were inductively analyzed using the thematic synthesis method [20]. This is a three-stage process beginning with the free, line-by-line coding of the findings extracted from the primary studies. The reviewers then searched for similarities and differences between the codes to categorize them into a hierarchical tree structure, from which descriptive themes were generated. The last step was to develop analytical themes. The reviewer interpreted the meaning of the themes and their associations with the research topic.

To ensure quality, memos were taken as part of the analysis process, and a matrix of synthesized findings regarding the interpretation was developed. Any disparities or discrepancies in coding were resolved through discussion or consultation with a third party, if necessary.

## 3. Results

### 3.1. Study Inclusion

**Figure 1:**
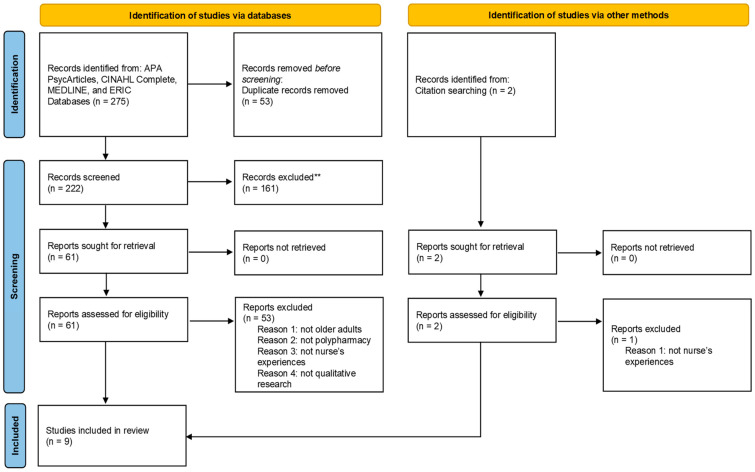
The PRISMA flow diagram illustrating the study selection process.

The screening process adhered to the Preferred Reporting Items for Systematic Reviews (PRISMA) protocol [21]. The flow diagram of the selection process is presented in Figure 1.

A primary search across five electronic databases yielded 275 records. After removing duplicates, 222 were screened for inclusion; 161 records were excluded based on their titles and abstracts. The remaining 61 records were retrieved and reviewed in full text for eligibility. After a full-text review, 8 records were eligible, and 53 were excluded according to the exclusion criteria. One record was identified through citation searching. 

### 3.2. Quality Assessment

All studies reported at least seven of the ten CASP checklist items. Most studies did not meet the criteria of adequately considering the relationship between researchers and participants. No study was excluded due to low quality. The quality assessment of the included studies is presented in Table 1. 

### 3.3. Characteristics of Included Studies

The key characteristics of the included studies are presented in Table 2. In these studies, the sample size ranged from 4 to 16, with 91 nurses from nine countries (Australia, Belgium, Canada, France, Sweden, Switzerland, Norway, the United Arab Emirates, and the United States). Three studies were conducted at comprehensive hospitals, two at primary health care centers, and six at long-term care facilities and nursing homes. Methods for data collection were semi-structured interviews (*n* = 4), focus group interviews (*n* = 3), interviews combined with observations (*n* = 1), and combined means of interviews (*n* = 1). Data were analyzed using thematic analysis (*n* = 3), content analysis (*n* = 1), the critical incident technique (*n* = 1), framework approaches (*n* = 1), grounded theory (*n* = 1), and systematic condensation (*n* = 1). One study used qualitative methods. 

### 3.4. Synthesized Findings 

The process of data analysis yielded four major descriptive themes: older adults suffering from polypharmacy, the importance of multidisciplinary teams, nursing roles in caring for older adults, and the complexity and barriers to implementing polypharmacy management. Each major theme was formed by several subthemes, which are supported by illustrative quotes. An overview of each major theme and subtheme is shown in Table 3, alongside quotes from each included study.

## 4. Discussion

### 4.1. Summary

This meta-analysis is the first work to examine existing findings regarding nurses’ experiences of polypharmacy in older adults. From nine included studies, we abstracted a plethora of nurses’ experiences and identified four interrelated syntheses related to: older adults’ daily interactions with polypharmacy, the significance of multidisciplinary teams, the role of nurses, and the complexity of and barriers to medication management. Based on these findings, several recommendations are proposed to improve care for older adults with polypharmacy. This study also suggests future research directions for understanding medication-related experiences and facilitating interventions within this population.

### 4.2. Comparisons with Existing Knowledge and Implications of Practice

The first theme we identified was that nurses recognize that polypharmacy is a common health concern among older adults which might impose burdens and challenges in their daily lives. This finding was consistent with the perceptions and beliefs of patients with multimorbidity [31,32], as well as the views of general practitioners [33]. Thus, consistent with prior quantitative evidence [34], health care professionals should understand patients’ burdens and be encouraged to facilitate in-person approaches and evidence-based practices to promote more effective interventions for polypharmacy.

In line with prior research [13], we found that nurses valued the importance of a multidisciplinary team and noted that they could benefit from collaboration, suggesting that full engagement in a team might be a key competency of nurses in caring for older adults with polypharmacy. A multidisciplinary team for polypharmacy facilitates collaboration between physicians, pharmacists, and other health care professionals. A previous retrospective study reported the efficacy of a multidisciplinary team in decreasing polypharmacy and potentially inappropriate medications [35]. In the intervention, team members evaluated the patient’s symptoms (e.g., physicians conducted physical and neurological examinations and nurses examined changes in symptoms and body function) and discussed the possibility of deprescribing. Each member examined the patient’s problems according to their expertise and skills. However, nurses also reported difficulties in working with other health care professionals. This is similar to past studies, which found that responsibilities for guaranteeing medication safety were unclear among health care professionals [36]. Thus, interprofessional team members should understand and clarify their responsibilities and procedures to reach a consensus about each competency when putting a multidisciplinary team into practice, especially in polypharmacy among older adults.

Many health care professionals feel powerless to manage and advocate for improved outcomes in patients living with polypharmacy due to a lack of formal training regarding this subject [36]. A plausible reason for this is that the definition of polypharmacy is varied in the literature, and this complexity makes the evaluation of polypharmacy and its association consequence difficult for those professionals [1]. We found that nurses reported such difficulties in understanding and managing polypharmacy when providing health care for older adults. In addition, nurses suggested that organizational factors might impede their work regarding polypharmacy [37]. Nursing is an essential part of health care services, and well-trained nurses can contribute to the health and well-being of patients [38]. As a result, education and training in the management of polypharmacy should address the diverse needs of nurses. Given that the ageing population is growing, future training for geriatric nurses might focus more attention on the knowledge, skills, and competencies of medication practice.

The past literature has outlined the irreplaceable role of nurses in medication management [39,40,41]. We found that nurses identified their roles at the individual level (as independent professionals) and the institutional level (as a link). However, physicians might propose that nurses play a minor role in medication management [40]. A survey on medication cessation among physicians showed that over one-third of physicians did not take nurses’ views about the discontinuation of medications seriously [42]. Essential education, previous experiences, and local laws might affect nurses’ role regarding medication management [43,44]. Therefore, other health care professionals should recognize the importance of nurses and their contributions to caring for patients with polypharmacy. Nurses should be encouraged to become involved in medication management and be empowered to take on more responsibilities.

### 4.3. Strengths and Limitations

We adopted four central criteria for identifying the strengths and limitations of this meta-synthesis: credibility, transferability, dependability, and confirmability [8,45].

We maintained and enhanced the credibility of our findings by searching, extracting, and analyzing qualitative data from different studies using a comprehensive and systematic review method. We also used the ENTREQ checklist to improve transparency in reporting this review.

The transferability of this review might be limited by several factors. First, the nurses in this review worked in various health care settings and had different academic and/or practical backgrounds. For example, half of the research settings were hospitals, and the rest were nursing homes where nursing staff might report distinct experiences concerning medication management. Next, the heterogeneity of the data describing nurses’ experiences and research settings may hinder an understanding of experiences across this geographical and ethnic region and within subgroups of the population. The countries in which studies were conducted tended to have higher incomes; therefore, data from middle- and low-income nations are lacking. Moreover, the subjective nature of our analysis might lead to a risk of bias. The varied definitions of polypharmacy used in each study might lead to increased heterogeneity of our findings. Readers should note that the findings of this review might provide in-depth perspectives of specific individuals and may not be representative of all nurses.

To ensure dependability, we examined the credibility of each finding based on the included studies and ensured that all the findings emerged from the original text. We also used a logical, three-stage process of thematic analysis and documented the research process.

A potential criticism impacting the confirmability of this synthesis is that the authors might influence the process of data extraction and synthesis. However, the authors of this study were experienced qualitative researchers. They participated in the analysis process and approved the presentation of major themes, subthemes, and illustrative quotes in this review. In addition, we have taken memos as part of the analysis process.

A strength of this study was that we used the CASP checklist to critically appraise the included studies. However, more than half of the studies did not identify the relationship between researcher and participants; thus, potential bias may occur. This meta-synthesis might have publication bias, as we only included studies published in English or Chinese and excluded grey literature such as conference abstracts.

### 4.4. Future Research

Evidence relating to nurses’ prescribing practices may not have emerged in all the included studies. Although researchers have demonstrated that nurses play a vital role in maintaining medications, jurisdictional solutions between nurses and medical professionals regarding prescribing work vary between countries. In addition, the competency of nurses in prescribing medications needs to be further examined.

There is a research gap in the engagement of key stakeholders in polypharmacy management for older adults. Nurses presented a variety of perspectives on the effectiveness of multidisciplinary teams in polypharmacy management. However, multiple layers of health care professionals (including general practitioners, nurses, pharmacists, etc.) are involved in the long-term care of elderly individuals. There are challenges in ensuring the participation and collaboration of all three specialties in actual practice.

The implementation of best practices regarding polypharmacy management across health care settings is important for promoting the health of older adults. Research should be undertaken to identify best practices and provide polypharmacy-related awareness and/or deprescription education to older adults and health care professionals. Additionally, the identification of barriers and enablers will help to develop and adopt best practices for older adults with polypharmacy.

## 5. Conclusions

Based on the limited studies available and the diversity of nursing standards globally, this meta-synthesis has uncovered four major themes regarding: the burden of polypharmacy, multidisciplinary teams, the role of nurses, and the complexity of medication management. We recommend that health care professionals be aware of the challenges and consequences of polypharmacy in older adults’ everyday lives and work to promote collaboration between various professionals, such as GPs, nurses, and pharmacists, in polypharmacy care plans. Multidisciplinary collaboration and communication between different health care professionals are essential for effective polypharmacy management. Nurses should be considered an essential element in the health care team when evaluating polypharmacy and medication management.

## Figures and Tables

**Table 1:** CASP Qualitative Studies Checklist *.

	Items of CASP Checklist	1 [22]	2 [23]	3 [24]	4 [25]	5 [26]	6 [27]	7 [28]	8 [29]	9 [30]
**1**	Was there a clear statement of the aims of the research?	Y	Y	Y	Y	Y	Y	Y	Y	Y
**2**	Is a qualitative methodology appropriate?	Y	Y	Y	Y	Y	Y	Y	Y	Y
**3**	Was the research design appropriate to address the aims of the research	Y	Y	Y	Y	Y	Y	Y	Y	Y
**4**	Was the recruitment strategy appropriate to the aims of the research	Y	Y	Y	Y	Y	Y	Y	Y	Y
**5**	Was the data collected in a way that addressed the research issue?	Y	Y	Y	Y	Y	Y	Y	Y	Y
**6**	Has the relationship between researcher and participants been adequately considered?	N	N	N	N	N	Y	Y	Y	N
**7**	Have ethical issues been taken into consideration?	Y	U	Y	Y	Y	Y	Y	Y	Y
**8**	Was the data analysis sufficiently rigorous?	U	U	Y	Y	Y	Y	Y	Y	Y
**9**	Is there a clear statement of findings?	Y	Y	Y	Y	Y	Y	Y	Y	Y
**10**	How valuable is the research?	Y	Y	Y	Y	Y	Y	Y	Y	Y

Y—Yes, N—No, U—Unclear, N/A—Not applicable; * All eligible studies are numbered chronologically.

**Table 2:** Key characteristics of included studies *.

Citation & Location	Objectives	Setting	Participants	Data Collection	Data Analysis	Key Findings	Limitations
Spinewine et al. (2005) Belgium [22]	To investigate the appropriateness of the use of medicines in older inpatients.	Acute wards	Four nurses (Female = two), aged 31–44 years	Interviews, focus groups, and observations	Grounded theory	This study identified three categories regarding inappropriate medication: relying on healthcare, passive perceptions regarding medication learning, and paternalistic decision-making.	Generalizability of data and researcher-participant interactions.
Skirbekk and Nortvedt (2014) Norway [23]	To investigate inadequate treatment for older adults from the views of healthcare professionals	Hospitals and general practice	Ten nurses (Female = six), aged 24–61 years	In-depth interviews and focus group interviews	Qualitative method	This study found that older patients were treated differently and some inadequate treatments were implemented.	Ethics considerations are not included and the analytic approach unclear.
Shemeili et al. (2016) the United Arab Emirates [24]	To investigate health professionals’ experiences regarding medication management in older adults	Hospitals	Seven nurses, age unknown	In-depth semi-structured, face-to-face interviews	Framework approach	This study identified six major aspects regarding medication management: Need for appropriate polypharmacy, systematic approach, communication and documentation, adherence, guidelines and policies, and multidisciplinary team.	Generalizability and single disease guideline not adjusted for multimorbidity.
Bell et al. (2017) Norway [25]	To investigate nurses’ learning experiences after participating in interprofessional medication reviews	Nursing home and home-based services units	Thirteen nurses, age and gender unknown	Semi-structured focus group interviews and telephone interviews	Systematic text condensation	This study described the five major themes: role in medication reviews, drug management, challenging the physician’s role, detailed information on patients, and patients’ symptoms and medication use.	Generalizability and a biased sample.
Sun et al. (2019) Canada [26]	To explore nurses’ experiences regarding barriers and enablers of deprescribing	Home care units	Eleven female nurses, aged 30–69	Focus group interviews	Thematic analysis	This study identified eight major themes of managing polypharmacy: reasons forpolypharmacy, challenges of managing polypharmacy, meaning of deprescribing, significance of deprescribing, barriers to awareness about deprescribing, facilitators to promote deprescribing, topics about deprescribing, and tools and resources concerning deprescribing.	Small sample size.
Foley et al. (2020) Swiss [27]	To compare practices and perceptions of nurses, pharmacists, and physicians regarding deprescribing	Nursing home	Ten nurses (Female = seven), age unknown	Focus group interviews	Content analysis	This study found that nurses focused on finding the right time, building trust, and considering purpose of stay at an individual level.	Generalizability and different data collection approaches used.
Pariseault et al. (2020) the United States [28]	To explore experiences of nursing practioners caring for older adults experiencing polypharmacy.	Primary healthcare	Fifteen nursing practioners (Female = fourteen), aged range 20–64 years	Semi-structured interviews	Thematic analysis	Four themes emerged: defining polypharmacy, communicating and collaborating, clinical judgement of nurse practitioners in relation to polypharmacy, and medication issues of older adults	Limited sample size.
Costa et al. (2021) France [29]	To explore healthcare professionals’ experiences regarding a novel telemediation review technology	Nursing home	Five nurses, age and gender unclear	Semi-structured interviews	Thematic analysis	This study identified four major themes: Healthcare professionals’ perceptions of the TMR, difficulties regarding medication management, healthcare professionals’ perceptions of the roles, and facilitators of best practice.	Small sample size and biased sample.
Holmqvist et al. (2021) Sweden [30]	To explore nurses’ experiences regarding the evaluations of older people’s medications	Primary healthcare center and home care units	Sixteen nurses (Female = fifteen), aged 26–61 years	Semi-structured interviews	Critical incident technique	This study identified the two aspects of medication evaluation including working in partnership and working conditions and two aspects of actions including working with a plan and working in collaborative way	Generalizability and loss of information regarding the incident.

* The studies below are presented in chronological order.

**Table 3:** An overview of the major theme, sub-theme, and illustrative quotes.

Major Theme	Sub-Theme	Distribution of the Main Theme	Illustrative Quotes
**Older adults suffering from polypharmacy**	Inappropriate medications	[22,23,24,26]	*Sometimes when we are talking with the patient and sometimes they will bring their medication. They have two bags of medication which—almost the same generic name but different brand name.* Page 111 [24]
	Relationship to medications	[26,27]	*“they have always obeyed to everything their physicians told them; they took everything they were told to take” (N8F).* Page 5 [27]
	Relationships with healthcare professionals	[22,23,26]	*I think that too often, they don’t ask what the patient thinks. For example, when a patient comes into hospital, they replace his laxative, X, by another laxative, Y. It mightn’t seem that important, but for the elderly person it is. Even just from a psychological point of view, I would say (nurse 3).* Page 3 [22]
**The importance of multidisciplinary team**	Communications between healthcare professionals	[24,27,28]	*…When it comes to the pharmacists, really we are not dealing with them, except if there is something that really needs to be addressed, we will call the pharmacy. (Nurse K1)* Page 111 [24]
	Learning and supporting each other	[25,28]	*The pharmacists gave us a very good impression by showing how much they could contribute regarding knowledge on drugs and drug therapy. They knew much more than we thought they did. Our previous impression was that they sold plasters and handled the drugs at the pharmacies. (Nurse, less than one year of experience with IMR* Page 4 [25]
	Relying on the multidisciplinary teams	[25,27,28]	*The pharmacist sees it from another angel and uses her own specialist knowledge to come up with new alternatives that the physician has not thought of–as far as I can see that must increase the quality.” (Nurse, with more than one year experience of IMR)* Page 5 [25]
**Nursing roles in caring for older adults**	As an independent healthcare professional	[24,29]	*“Well, I take care of the medicine trolleys, receiving medications [ie, from the pharmacy], I check if they’ve given the right medication, I adjust them according to treatment changes. Today for example, there have been a lot of treatment changes.”* Page 26 [29]
	As a link in a team	[27,30]	*You know, she [the patient] has so many contacts with health services overall, so I kind of end up in between all these contacts, you could say. [Person10]* Page 4 [30]
**The complexity and barriers of implementing polypharmacy management**	Complex in management	[25,26,28]	*“We have learned more about combination of different drugs and anticholinergic effects. (…) Being more aware on pain relief–the need to assess the treatment more often and at an earlier stage. Previously they had Paracetamol 1 g × 3 without us assessing, but now we ask them whether they still need them. The questions pop up more frequently.” (Nurse, more than one year experience of IMR)* Page 6 [25]
	Lack of source	[26,28,29]	*When I got a referral that the patient was complaining about dizziness, I made a home visit and found out that they were on high dosages of anti-hypertensive… I have been communicating with the doctor to adjust the level of this medication. (FG1, P1)* *Another participant added that medications are often being prescribed without proper evaluation or follow-up to assess for the appropriateness of the medication regimen.* *When one medication is not successful, they (the doctors or nurse practitioners) added on something else instead of just working through and figuring out which medication is the most appropriate for that particular client. (FG1, P5)* Page 4 [26]
	Fragmented healthcare	[27,28]	*“We have to work with 10 different physicians who don’t have a common philosophy. Not all physicians will have the same attitude. They all come on their own time, (…) once they have seen all the patients, at six in the evening, and we are pretty busy at that time.” (N9F)* Page 10 [27]

## Data Availability

The datasets generated during and/or analyzed during the current study are available from the corresponding author on reasonable request.

## References

[1] Masnoon N., Shakib S., Kalisch-Ellett L., Caughey G.E. (2017). What is polypharmacy? A systematic review of definitions. BMC Geriatr..

[2] Chen C., Feng Z., Fu Q., Wang J., Zheng Z., Chen H., Feng D. (2021). Predictors of polypharmacy among elderly patients in China: The role of decision involvement, depression, and taking Chinese medicine behavior. Front. Pharmacol..

[3] Young E.H., Pan S., Yap A.G., Reveles K.R., Bhakta K. (2021). Polypharmacy prevalence in older adults seen in United States physician offices from 2009 to 2016. PLoS ONE.

[4] Midão L., Giardini A., Menditto E., Kardas P., Costa E. (2018). Polypharmacy prevalence among older adults based on the survey of health, ageing and retirement in Europe. Arch. Gerontol. Geriatr..

[5] Dumbreck S., Flynn A., Nairn M., Wilson M., Treweek S., Mercer S.W., Alderson P., Thompson A., Payne K., Guthrie B. (2015). Drug-disease and drug-drug interactions: Systematic examination of recommendations in 12 UK national clinical guidelines. BMJ.

[6] Hand B.N., Krause J.S., Simpson K.N. (2018). Polypharmacy and adverse drug events among propensity score matched privately insured persons with and without spinal cord injury. Spinal Cord.

[7] Pazan F., Wehling M. (2021). Polypharmacy in older adults: A narrative review of definitions, epidemiology and consequences. Eur. Geriatr. Med..

[8] Eriksen C.U., Kyriakidis S., Christensen L.D., Jacobsen R., Laursen J., Christensen M.B., Frølich A. (2020). Medication-related experiences of patients with polypharmacy: A systematic review of qualitative studies. BMJ Open.

[9] Kim J., Parish A.L. (2017). Polypharmacy and medication management in older adults. Nurs. Clin. N. Am..

[10] Mardani A., Griffiths P., Vaismoradi M. (2020). The role of the nurse in the management of medicines during transitional care: A systematic review. J. Multidiscip. Healthc..

[11] Yang C., Lee D.T.F., Wang X., Chair S.Y. (2022). Effects of a nurse-led medication self-management intervention on medication adherence and health outcomes in older people with multimorbidity: A randomised controlled trial. Int. J. Nurs. Stud..

[12] Doyle L., McCabe C., Keogh B., Brady A., McCann M. (2020). An overview of the qualitative descriptive design within nursing research. J. Res. Nurs..

[13] Farrell B., Thompson W., Black C.D., Archibald D., Raman-Wilms L., Grassau P., Patel T., Weaver L., Eid K., Winslade N. (2018). Health care providers’ roles and responsibilities in management of polypharmacy: Results of a modified Delphi. Can. Pharm. J..

[14] Köberlein J., Gottschall M., Czarnecki K., Thomas A., Bergmann A., Voigt K. (2013). General practitioners’ views on polypharmacy and its consequences for patient health care. BMC Fam. Pract..

[15] Hansen C.R., Byrne S., O’Mahony D., Kearney P.M., Sahm L.J. (2019). Qualitative analysis of community pharmacists’ opinions on their involvement in reducing potentially inappropriate prescribing. Eur. J. Clin. Pharmacol..

[16] Rowe S., Pittman N., Balsom C., Druken R., Kelly D.V. (2022). Beliefs and attitudes of residents, family members and healthcare professionals regarding deprescribing in long-term care: A qualitative study. Int J Clin Pharm.

[17] Danielsen B.V., Sand A.M., Rosland J.H., Førland O. (2018). Experiences and challenges of home care nurses and general practitioners in home-based palliative care-a qualitative study. BMC Palliat. Care.

[18] Tong A., Flemming K., McInnes E., Oliver S., Craig J. (2012). Enhancing transparency in reporting the synthesis of qualitative research: ENTREQ. BMC Med. Res. Methodol.

[19] Critical Appraisal Skills Programme CASP qualitative studies checklist. https://casp-uk.net/casp-tools-checklists/.

[20] Thomas J., Harden A. (2008). Methods for the thematic synthesis of qualitative research in systematic reviews. BMC Med. Res. Methodol..

[21] Page M.J., McKenzie J.E., Bossuyt P.M., Boutron I., Hoffmann T.C., Mulrow C.D., Shamseer L., Tetzlaff J.M., Akl E.A., Brennan S.E. (2020). The PRISMA 2020 statement: An updated guideline for reporting systematic reviews. BMJ.

[22] Spinewine A., Swine C., Dhillon S., Franklin B.D., Tulkens P.M., Wilmotte L., Lorant V. (2005). Appropriateness of use of medicines in elderly inpatients: Qualitative study. BMJ.

[23] Skirbekk H., Nortvedt P. (2014). Inadequate treatment for elderly patients: Professional norms and tight budgets could cause “ageism” in hospitals. Health Care Anal..

[24] Al Shemeili S., Klein S., Strath A., Fares S., Stewart D. (2016). An exploration of health professionals’ experiences of medicines management in elderly, hospitalised patients in Abu Dhabi. Int. J. Clin. Pharm..

[25] Bell H.T., Granas A.G., Enmarker I., Omli R., Steinsbekk A. (2017). Nurses’ and pharmacists’ learning experiences from participating in interprofessional medication reviews for elderly in primary health care - A qualitative study. BMC Fam. Pract..

[26] Sun W., Tahsin F., Barakat-Haddad C., Turner J.P., Haughian C.R., Abbass-Dick J. (2019). Exploration of home care nurse’s experiences in deprescribing of medications: A qualitative descriptive study. BMJ Open.

[27] Foley R.A., Hurard L.L., Cateau D., Koutaissoff D., Bugnon O., Niquille A. (2020). Physicians’, nurses’ and pharmacists’ perceptions of determinants to deprescribing in nursing homes considering three levels of action: A qualitative study. Pharmacy.

[28] Pariseault C.A., Sharts-Hopko N., Blunt E. (2020). Nurse practitioners’ experiences of polypharmacy in community-dwelling older adults. J. Am. Assoc. Nurse Pract..

[29] Costa M., Correard F., Montaleytang M., Baumstarck K., Loubière S., Amichi K., Villani P., Honore S., Daumas A., Verger P. (2021). Acceptability of a novel telemedication review for older adults in nursing homes in France: A qualitative study. Clin. Interv. Aging.

[30] Holmqvist M., Thor J., Ros A., Johansson L. (2021). Evaluation of older persons’ medications: A critical incident technique study exploring healthcare professionals’ experiences and actions. BMC Health Serv. Res..

[31] Smith A., Macaden L., Kroll T., Alhusein N., Taylor A., Killick K., Stoddart K., Watson M. (2019). A qualitative exploration of the experiences of community dwelling older adults with sensory impairment/s receiving polypharmacy on their pharmaceutical care journey. Age Ageing.

[32] Mohammed M.A., Moles R.J., Chen T.F. (2016). Medication-related burden and patients’ lived experience with medicine: A systematic review and metasynthesis of qualitative studies. BMJ Open.

[33] Anthierens S., Tansens A., Petrovic M., Christiaens T. (2010). Qualitative insights into general practitioners views on polypharmacy. BMC Fam. Pract..

[34] McNeil M.J., Kamal A.H., Kutner J.S., Ritchie C.S., Abernethy A.P. (2016). The burden of polypharmacy in patients near the end of life. J. Pain. Symptom. Manag..

[35] Seto H., Ishimaru N., Ohnishi J., Kanzawa Y., Nakajima T., Shimokawa T., Imanaka Y., Kinami S. (2022). Multidisciplinary team deprescribing intervention for polypharmacy in elderly orthopedic inpatients: A propensity score-matched analysis of a retrospective cohort study. Intern. Med..

[36] Syyrilä T., Vehviläinen-Julkunen K., Härkänen M. (2021). Healthcare professionals’ perceptions on medication communication challenges and solutions-text mining and manual content analysis-cross-sectional study. BMC Health Serv. Res..

[37] Høghaug G., Skår R., Tran T.N., Schou-Bredal I. (2019). Nurses’ experiences with newly acquired knowledge about medication management: A qualitative study. J. Nurs. Manag..

[38] Coster S., Watkins M., Norman I.J. (2018). What is the impact of professional nursing on patients’ outcomes globally? An overview of research evidence. Int. J. Nurs. Stud..

[39] Choo J., Hutchinson A., Bucknall T. (2010). Nurses’ role in medication safety. J. Nurs. Manag..

[40] Huisman B.A.A., Geijteman E.C.T., Dees M.K., Schonewille N.N., Wieles M., van Zuylen L., Szadek K.M., van der Heide A. (2020). Role of nurses in medication management at the end of life: A qualitative interview study. BMC Palliat. Care.

[41] Rohde E., Domm E. (2018). Nurses’ clinical reasoning practices that support safe medication administration: An integrative review of the literature. J. Clin. Nurs..

[42] Geijteman E.C.T., Huisman B.A.A., Dees M.K., Perez R., van der Rijt C.C.D., van Zuylen L., van der Heide A. (2018). Medication discontinuation at the end of life: A questionnaire study on physicians’ experiences and opinions. J. Palliat. Med..

[43] Wilson E., Morbey H., Brown J., Payne S., Seale C., Seymour J. (2015). Administering anticipatory medications in end-of-life care: A qualitative study of nursing practice in the community and in nursing homes. Palliat. Med..

[44] Maier C.B. (2019). Nurse prescribing of medicines in 13 European countries. Hum. Resour. Health.

[45] Lincoln Y.S., Guba E.G. (1985). Naturalistic Inquiry.

